# Real-World Treatment Patterns and Clinical Outcomes Among Patients with Metastatic Renal Cell Carcinoma Post-Immune-Oncology and Vascular Endothelial Growth Factor Receptor Targeted Therapies †

**DOI:** 10.3390/cancers17091434

**Published:** 2025-04-25

**Authors:** Neil J. Shah, Sneha Sura, Reshma Shinde, Junxin Shi, Manojkumar Bupathi, Donna Vickery, Rodolfo Perini, Robert J. Motzer

**Affiliations:** 1 Memorial Sloan Kettering Cancer Center, New York, NY 10065, USA; motzerr@mskcc.org; 2 Weill Corneal Medical Hospital, New York, NY 10065, USA; 3 Ontada, Boston, MA 02110, USA; sneha.sura@mckesson.com (S.S.); junxin.shi@mckesson.com (J.S.); manojkumar.bupathi@usoncology.com (M.B.); 4 Merck & Co., Inc., Rahway, NJ 07065, USA; reshma.shinde@merck.com (R.S.); donna.vickery@merck.com (D.V.); rodolfo.perini@merck.com (R.P.)

**Keywords:** metastatic renal cell carcinoma, immune-oncology, tyrosine kinase inhibitors, progression-free survival, overall survival

## Abstract

Background: The treatment landscape of metastatic renal cell carcinoma (mRCC) has evolved rapidly with the introduction of various immune-oncology (IO) agents and tyrosine kinase inhibitors (TKIs). We aimed to describe real-world treatment patterns and clinical outcomes of mRCC patients in post-IO and TKI settings. Methods: Using data from The US Oncology Network electronic health record database, iKnowMed, this retrospective cohort study included adult mRCC patients receiving subsequent treatments (index treatment) post-IO and TKI in combination or sequence between 1 January 2018 and 30 September 2020 and followed them until 30 April 2022. Treatment patterns were summarized descriptively. Overall survival (OS) and progression-free survival (PFS) from the index date was described using Kaplan–Meier analysis. Results: We identified 239 patients diagnosed with mRCC who received anticancer treatment post-IO and TKI therapies. The median age was 67 (range: 58, 73) years, 73.6% were male, and 61.5% had an intermediate/poor International Metastatic Renal Cell Carcinoma Database Consortium risk score. Among these, 29 (12.1%) received subsequent therapy at the line of therapy 2 (LOT2), 167 (69.8%) at LOT3, and 43 (18.0%) at LOT4+. The most common subsequent treatments were cabozantinib (38.5%) and axitinib (10.5%). The median OS for LOT2, LOT3, and LOT4+ was 18.0, 17.0, and 26.9 months, respectively. The median PFS for LOT2, LOT3, and LOT4+ was 6.1, 5.0, and 4.0 months, respectively. Conclusions: In mRCC patients treated with IO and TKI in combination/sequence, subsequent LOT choice is diverse, with TKI-based treatments being the most preferred. There is a need for considering treatment sequencing studies and studies with a new mode of action in this population.

## Simple Summary

Recent advancements in treating metastatic kidney cancer have led to new options for patients who have already received treatments like immunotherapy or tyrosine kinase inhibitors. This study looked at real-world data from the US Oncology Network to see what treatments are commonly used after these initial therapies and how well they work. The most common treatments after immunotherapy or tyrosine kinase inhibitors were cabozantinib and axitinib. These treatments helped patients live longer, with some living between 18 to 26 months. Additionally, cancer progression was slowed for about 4 to 6 months for most patients. However, this study showed that there was not a big difference in survival between different treatments. This means that while these treatments are helpful, there is still a lot of room for improvement. New and better treatment options are needed to help patients more effectively.

## 1. Introduction

Renal cell carcinoma (RCC) represents the most common form of kidney cancer, accounting for approximately 90% of all kidney cancer malignancies. The incidence of RCC has been rising globally, with an estimated 81,610 new cases and 14,390 deaths expected in the United States (US) in 2024 [1]. Survival varies by stage of disease, with a 5-year relative survival rate of 93.3% for localized disease and 18.2% for distant metastatic disease [2]. At the time of initial diagnosis, 13% of patients with RCC were found to have metastatic disease (mRCC) [3].

Over the past decade, the advent of immune-oncology (IO) agents and vascular endothelial growth factor tyrosine kinase inhibitors (VEGF-TKIs) have transformed the treatment landscape for mRCC [4,5,6]. These agents are the new standard of care first-line (1L) treatments based on robust clinical trial data [7,8,9,10]. Limited data exist regarding subsequent line of treatments post-modern IO-IO or IO-TKI combination treatments in the 1L setting [11]. Recently, the CONTACT-03 trial investigated the combination of atezolizumab (an IO agent) with cabozantinib (a TKI) vs. cabozantinib monotherapy in mRCC patients who had progressed on prior IO therapy and noted no difference between both treatment groups [12]. Furthermore, a recent TiNivo-2 study also noted similar findings with no difference between tivozanib plus nivolumab vs. tivozanib monotherapy [13]. Thus, the role of IO-based regimens in post-IO setting seems to be limited. Several other TKI-based regimens have shown striking efficacy in prior TKI-treated patients, including cabozantinib (METOR) [14], lenvatinib plus everolimus [15], tivozanib (TIVO-3) [16], and most recently, belzutifan (LITESPARK-005) [17]. These agents are now FDA-approved for subsequent lines of treatment for mRCC patients. Given that the National Comprehensive Cancer Network (NCCN) guidelines have no recommended regimens as a preferred category for subsequent lines of treatments [11], and other recommended regimen categories include cabozantinib, tivozanib, belzutifan, lapatinib plus everolimus, axitinib, and IO+TKI combinations (for IO-naïve patients), the sequencing of these agents and efficacy, especially in the real-world setting, remain obscure [18,19,20,21,22,23,24,25,26,27].

Overall, the field of kidney cancer is rapidly evolving, especially with the approval of IO agents and VEGF-TKIs. Several factors influence these treatment choices, including the patient’s comorbidities, performance status, toxicities from prior treatments, insurance status, drug availability, and physician preferences. Limited data exist regarding subsequent treatments sequencing, and clinical outcomes in mRCC patients receiving IO and VEGF-TKIs, especially in real-world settings. Hence, we characterize the treatment patterns and clinical outcomes of mRCC patients who received subsequent systemic anticancer therapies after IO agents and VEGF-TKIs in real-world community oncology settings.

## 2. Methods

### 2.1. Data Source

Study data were sourced from The US Oncology Network’s electronic health record (EHR) system, iKnowMed (iKM). The US Oncology Networks comprises over 600 community-based outpatient practices across 31 states, and iKM includes over 1.4 million patients treated annually [28]. The Social Security Administration’s Death Master File was used to supplement the data available in iKM on vital status and death dates. The resulting structured data were augmented with unstructured data abstracted from medical charts to capture information of interest not otherwise available from structured fields in the EHR. This included patients’ treatment history, date of diagnosis, and physician-assessed response. The abstracted data were collected using a secure, web-based electronic report form by healthcare professionals with oncology experience under standardized guidelines. The US Oncology Inc. Institutional Review Board granted this study an exception and waiver of consent.

### 2.2. Study Design and Population

This retrospective study included adult patients diagnosed with mRCC receiving a subsequent treatment in any line of therapy (LOT) for up to 3 LOTs, after being treated with IO and TKI in combination or sequence between 1 January 2018 and 30 September 2020 (study identification period). These also included patients who received treatments post-IO-IO followed by TKI or IO+TKI. The index date was defined as the date of initiation of subsequent treatments post-IO and TKI. Patients’ records were followed through 30 April 2022 (end of the study observation period), death, or last contact date, whichever occurred first. Additionally, patients were required to have at least 2 visits within The US Oncology Network clinics during the study observation period to be included in the study. Patients were excluded if their EHRs were inaccessible for research purposes, enrolled in an interventional clinical trial, or received treatment indicated for another primary cancer during the study observation period.

### 2.3. Study Variables

Patient characteristics: Demographic characteristics included age, gender, and race. Clinical characteristics included Eastern Cooperative Oncology Group (ECOG) Performance Status (PS), histology, and the International mRCC Database Consortium (IMDC) risk score. These characteristics were assessed at or closest to the index date.

Treatment sequencing: Patients’ treatment sequences were considered based on the absolute order of regimens, as determined by start and stop dates. Drugs administered within 28 days of the current treatment were considered a combination therapy.

Clinical outcomes: Clinical effectiveness was assessed using overall survival (OS) and progression-free survival (PFS). OS was defined as the interval between the index date and the date of death. Patients who did not die were censored on their last contact date or study end date. PFS was defined as the interval between the index date and clinician-documented disease progression or death date. Patients who did not progress or die were censored on their last contact date or study end date.

### 2.4. Statistical Analysis

Patients’ demographic and clinical characteristics and treatment patterns were summarized using descriptive statistics. Clinical outcomes were stratified by LOT as well as by individual index regimen (with sample size ≥ 10). Continuous variables were summarized using mean, standard deviation (SD), median, and interquartile range (IQR). Categorical variables were described using counts and percentages. Time-to-event outcomes such as OS and PFS were analyzed using Kaplan–Meier methods. The Cox proportional hazard regression model assessed the association between individual index regimens with OS and PFS. The models were adjusted for age, gender, race, ECOG PS, histology, and IMDC risk score. These covariates were chosen based on their statistical and clinical significance. We used the indicator variable for the missing data in the regression analysis. All the analyses were conducted using SAS v.9.4 with a priori significance level set for *p*-value < 0.05.

## 3. Results

After applying eligibility criteria, 239 mRCC patients were included in the analysis. The median (IQR) age of the study cohort was 67 (58, 73) years, with the majority being male (73.6%) and Caucasian (77.0%). Most patients (85.4%) had clear cell histology, 63.2% had ECOG 0–1, and 61.5% had intermediate/poor IMDC risk score. The median (IQR) follow-up time was 14.4 (5.5, 23.7) months. Patient characteristics by index LOT are presented in Table 1.

**Figure 1:**
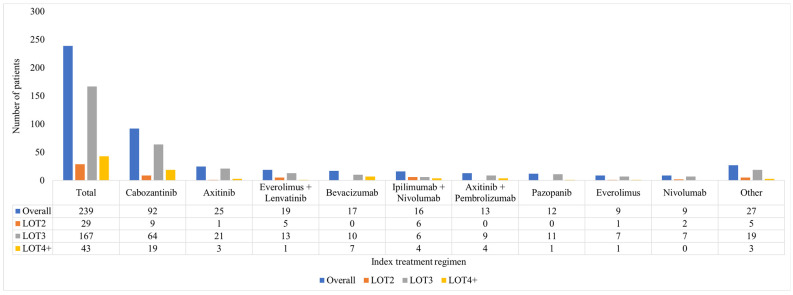
Patients diagnosed with mRCC initiating subsequent treatment post-IO and TKI-based therapy, index treatment by LOT. Abbreviations: IO—immune-oncology; LOT—line of therapy, mRCC—metastatic renal cell carcinoma; TKI—tyrosine kinase inhibitor; other treatments include temsirolimus (*n* = 7), cabozantinib + nivolumab (*n* = 5), sunitinib (*n* < 5), avelumab +axitinib (*n* < 5), axitinib + nivolumab (*n* < 5), lenvatinib + pembrolizumab (*n* < 5), bevacizumab + irinotecan (*n* < 5), pazopanib + pembrolizumab (*n* < 5), and sorafenib (*n* < 5).

Overall, 29 (12.1%) received treatment in LOT2, 167 (69.9%) in LOT3 and 43 (18.0%) in LOT4+. The most common treatments post-IO and TKI-based therapy were TKI monotherapies (53.8%), including cabozantinib (38.5%), and axitinib (10.5%), Everolimus + lenvatinib (7.9%), bevacizumab (7.1%), and ipilimumab + nivolumab (6.7%) were also used (Figure 1).

**Figure 2:**
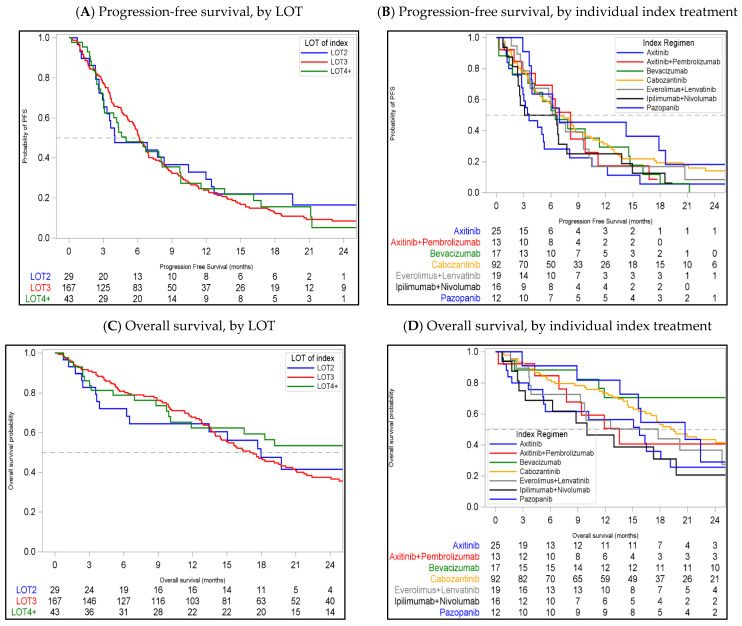
Clinical outcomes among patients receiving subsequent treatments post IO and TKI-based therapies, by LOT and individual index treatments.

The median (95% CI) PFS for LOT2, LOT3, and LOT4+ was 4.0 (2.9, 12.0) months, 6.1 (5.6, 6.9), and 5.0 (3.0, 8.1) with 12-month PFS probability (95% CI) of 29.3% (14.0, 46.5), 24.0% (17.6, 30.9), and 24.6% (12.6, 38.7), respectively (Table 2 and Figure 2A). The median (95% CI) PFS was 7.0 (5.1, 8.7) months for cabozantinib, 3.6 (2.8, 5.3) months for axitinib, 6.7 (3.6, 9.6) months for everolimus + lenvatinib, 6.5 (1.7, 14.6) months for bevacizumab, and 4.9 (2.3, 7.8) months for ipilimumab + nivolumab. The median PFS for all the index treatments is described in Table 3 and Figure 2B.

The median (95% CI) OS for LOT2, LOT3, and LOT4+ was 18.0 (6.2, NR), 17.0 (14.4, 20.7), and 26.9 (10.1, 30.1) months with 12-month survival probability (95% CI) of 60.5% (39.0, 76.5), 69.5% (61.8, 76.1), and 68.8% (40.5, 85.6), respectively (Table 2 and Figure 2C). The median (95% CI) OS was 19.5 (16.4, 26.9) months for cabozantinib, 15.7 (5.2, 19.1) months for axitinib, 17.8 (3.9, 35.8) months for everolimus + lenvatinib, 30.3 (11.3, NR) months for bevacizumab, and 10.0 (2.6, 19.7) months for ipilimumab + nivolumab. The median OS for all the index treatments is described in Table 3 and Figure 2D.

Table 2, Table 3 and Table S1 describe the univariable and multivariable analysis for PFS and OS. In the multivariable regression analysis, no statistically significant difference in OS was observed across the different index treatments or LOT. Similar results were observed for PFS for LOT and across various index treatments, except a shorted PFS for axitinib compared to cabozantinib (adjusted HR: 1.79 [95% CI: 1.02–3.15]).

## 4. Discussion

The approval of various IO agents and VEGF-TKIs has revolutionized front-line treatment in mRCC patients, affecting the choice of subsequent therapies. This real-world study characterized treatment patterns and clinical outcomes of 239 mRCC patients who received subsequent anticancer treatment post-IO and TKI-based therapies in community oncology settings. Our study identified a range of treatments utilized in these settings, including TKIs, IO, mTOR inhibitors, IO + TKI, and TKI+mTOR. The most frequently used treatments were TKI-based, with cabozantinib being the most common agent, followed by axitinib and a combination of everolimus plus lenvatinib. Additionally, our findings indicated that the choice of a specific treatment following IO and TKI based therapies did not have an impact on OS and PFS.

The optimal treatment sequencing following IO and TKIs in mRCC remains poorly defined. While the number of available therapy options is increasing, there is ongoing debate about the best approach to treatment sequencing. Most clinical trials emphasize newer therapies rather than the sequencing of treatments, leaving physicians with limited guidance for selecting therapies in later LOTs. Our study highlighted the heterogeneity in subsequent LOTs, as we observed a wide variety of treatment selections after IO and TKI, regardless of the LOT. TKI-based treatments remained the most common choice across all LOTs. We also noted that many patients received combinations such as ipilimumab plus nivolumab, axitinib plus pembrolizumab, cabozantinib plus nivolumab, and lenvatinib plus pembrolizumab in LOTs 2–4, despite the negative randomized controlled trial for such approaches [12,13].

Our study noted that patients receiving treatments in LOT4+ had a numerically higher median overall survival (OS) of 26.9 months, compared to 18.0 months for LOT2 and 17.0 months for LOT3. However, their median progression-free survival (PFS) was numerically shorter at 4.0 months, as opposed to 5.0 months for LOT2 and 6.1 months for LOT3. This suggests that patients who can access treatments in later lines of therapy (LOT) may have different biological profiles and are likely to experience prolonged survival and better disease control. These findings align with the results of the TIVO-3 trial, which indicated prolonged survival with both tivozanib and sorafenib, showing OS figures of 16.4 months for tivozanib and 19.1 months for sorafenib (*p* = 0.353) in patients who had previously failed at least two systemic therapies, with at least one prior treatment being a tyrosine kinase inhibitor (TKI) [16,29]. The results of both studies are likely influenced by patients’ biological risk factors and the biology of mRCC. Patients who are fit, with fewer comorbidities, are more likely to receive treatments in latter LOTs and may also declare themselves as having indolent disease, which is typically associated with better prognoses.

Our study found a median OS of 19.5 months and PFS of 7.0 months for cabozantinib. This is consistent with the METEOR trial, which reported a median PFS of 7.4 months and an OS of 21.4 months [14,22]. For the combination of everolimus + lenvatinib, we observed a median PFS of 6.7 months and a median OS of 17.8 months. This was inferior to a previously reported phase 2 study of everolimus + Lenvatinib, which showed a median PFS of 14.6 months and a median OS of 25.4 months [20]. The differences between these studies may be attributed to variations in the patient populations and the usability/tolerability of this combination in real-world settings. Overall, we did not observe any difference in PFS or OS among various, which included cabozantinib, everolimus + lenvatinib, axitinib, axitinib, pazopanib, and ipilimumab + nivolumab for PFS or OS. Notably, we observed a numerically lower OS for ipilimumab + nivolumab. The findings from our study suggest that the treatment landscape after IO and TKI therapies are complex, with no difference in efficacy outcomes among various treatments.

The treatment landscape for mRCC is rapidly evolving. Two recent studies, CONTACT-03 and TiNivo-2, have evaluated the roles of IO and TKI agents in clear cell mRCC patients who have already received IO therapy. The CONTACT-03 trial compared the combination of atezolizumab and cabozantinib with cabozantinib alone in clear cell mRCC patients who progressed after prior IO therapy. In this trial, 67% of the patients received a TKI before their IO therapy. The progression-free survival (PFS) for the combination therapy was comparable to that of cabozantinib alone, at 10.6 months versus 10.8 months, respectively (*p* = 0.78) [12]. Similarly, the TiNivo-2 trial assessed the combination of tivozanib and nivolumab against tivozanib alone. The results showed similar PFS rates for both treatments, with 5.7 months for the combination and 7.4 months for tivozanib alone (*p* = 0.49) [13]. Both trials indicated a limited effectiveness of IO-based treatments for mRCC patients who had progressed after previous IO treatments. More recently, belzutifan, a novel hypoxia-inducible factor-2 alpha (HIF-2a) inhibitor, has been approved based on results from the LITESPARK-005 study. This trial evaluated the efficacy of belzutifan compared to everolimus in patients with clear cell mRCC who had previously progressed on both IO and TKI therapies. Results showed that patients receiving belzutifan had improved PFS (HR (95% CI) = 0.74 (0.63,0.88, *p* = 0.00031) [17]. Given the rapid development of the mRCC space, especially post-IO and TKI settings, our study provides critical insights into translating these advances into real-world community settings.

This is a retrospective real-world evidence study, so limitations pertaining to the use of retrospective data apply to this study, including the observational nature of the study and unmeasured cofounding, as well as limitations for causality assessment. Treatment selection in the real world can be influenced by patient characteristics, treatment availability, physician preference, etc. The data collected by the iKM EHR were not created solely for research purposes; therefore, the data standardization collection methods and instruments could be influenced by physician reporting practices. Additionally, not all practices utilize the full capabilities of the iKM EHR, which may have resulted in selection bias. Practices within The US Oncology Network may differ from other oncology practices in the composition of their patient population or the prescribing practices of their physicians; this may limit generalizability to other oncology settings. Patients eligible for this study had to survive from the mRCC diagnosis date to the start of the index LOT, potentially introducing immortal time bias. Future retrospective studies should use landmark survival analyses or time-dependent Cox regression analyses. The study follow-up ended in 2022, and since then, the FDA approved belzutifan for mRCC post-IO and TKI treatment, warranting further research on its impact on prescribing practices.

## 5. Conclusions

This is among the first real-world studies conducted within well-organized and geographically dispersed community oncology practices, providing valuable insights into treatment patterns and clinical outcomes in patients receiving treatment after IO and TKI-based therapies A substantial heterogenicity exists in treatment selection in this space with TKI-based treatments, such as cabozantinib and axitinib, being the most commonly used. Individual treatments post-IO and TKI showed similar clinical benefits for OS and PFS. There is a need for considering treatments with newer modes of action in this population. Future studies with a larger sample size can further validate these findings.

## Figures and Tables

**Table 1:** Demographic and clinical characteristics of patients initiating subsequent treatment post-IO and TKI-based treatment, by LOTs.

Patient Characteristics	Overall Study Population (*n* = 239)	Line of Therapy (LOT)		
		LOT2 (*n* = 29)	LOT3 (*n* = 167)	LOT4+ (*n* = 43)
Median age, years (IQR)	67 (58, 73)	65 (58, 70)	67 (60, 73)	68 (55, 72)
Age group, *n* (%)				
<65	100 (41.8)	14 (48.3)	68 (40.7)	18 (41.9)
≥65 years	139 (58.2)	15 (51.7)	99 (59.3)	25 (58.1)
Gender, *n* (%)				
Female	63 (26.4)	10 (34.5)	46 (27.5)	7 (16.3)
Male	176 (73.6)	19 (65.5)	121 (72.5)	36 (83.7)
Race, *n* (%)				
White or Caucasian	184 (77.0)	20 (69.0)	126 (75.4)	38 (88.4)
Other race	14 (5.9)	<5	11 (6.6)	<5
Not documented	26 (10.9)	8 (27.6)	17 (10.2)	<5
ECOG, *n* (%)				
0–1	151 (63.2)	13 (44.8)	106 (63.5)	32 (74.4)
2+	32 (13.4)	4 (13.8)	22 (13.2)	6 (14.0)
Not documented	56 (23.4)	12 (41.4)	39 (23.4)	5 (11.6)
Histology, *n* (%)				
Clear cell	204 (85.4)	21 (72.4)	144 (86.2)	39 (90.7)
Non-clear cell	13 (5.4)	<5	7 (4.2)	<5
Other	21 (8.8)	<5	15 (9.0)	<5
Not documented	13 (5.4)	<5	7 (4.2)	<5
IMDC risk score, *n* (%)				
Favorable/Intermediate	81 (33.9)	<5	61 (25.5)	18 (7.5)
Intermediate/Poor	147 (61.5)	25 (10.5)	99 (41.4)	23 (9.6)
Not documented	11 (4.6)	<5	7 (2.9)	<5
Median time from mRCC diagnosis to initiation of index treatment, days (IQR)	631 (356.0, 1219.0)	228 (175.0, 339.0)	632 (370.0, 1178.0)	1242 (647.0, 1694.0)
Median follow-up, months (IQR)	14.4 (5.5, 23.7)	13.7 (3.6, 19.7)	14.4 (6.1, 23.4)	16 (5.3, 27.4)

Abbreviations: BMI—body mass index; ECOG—eastern cooperative oncology group; IMDC—international metastatic renal cell carcinoma database consortium; IQR—interquartile range; LOT—line of therapy; Max—maximum; Min—minimum; mRCC—metastatic renal cell carcinoma; and SD—standard deviation.

**Table 2:** Association of LOT with clinical outcomes, results from KM and adjusted Cox regression models.

Clinical Outcomes	Overall Study Population (*n* = 239)	Line of Therapy (LOT)		
		LOT2 (*n* = 29)	LOT3 (*n* = 167)	LOT4+ (*n* = 43)
Real-world progression-free survival				
Median (95% CI)	6.1 (5.1, 6.8)	4.0 (2.9, 12.0)	6.1 (5.6, 6.9)	5.0 (3.0, 8.1)
12-month PFS probability, % (95% CI)	24.8% (19.3, 30.6)	29.3% (14.0, 46.5)	24.0% (17.6, 30.9)	24.6% (12.6, 38.7)
Multivariable analysis, Adjusted HR	-	Reference	1.25 (0.75–2.08)	1.36 (0.74–2.51)
Overall survival				
Median (95% CI)	17.8 (15.0, 21.0)	18.0 (6.2, NR)	17.0 (14.4, 20.7)	26.9 (10.1, 30.1)
12-month OS probability, % (95% CI)	66.4% (59.8, 72.2)	64.5% (44.0, 79.1)	67.8% (59.8, 74.5)	62.4% (45.3, 75.5)
Multivariable analysis, Adjusted HR	-	Reference	1.32 (0.75–2.33)	1.36 (0.65–2.83)

Abbreviations: CI—confidence interval; KM—Kaplan-Meier; LOT—line of therapy; NR—not reached; OS—overall survival; PFS—progression-free survival.

**Table 3:** Association of index treatments with clinical outcomes, Results from KM and adjusted Cox regression models.

Clinical Outcomes	Cabozantinib (*n* = 92)	Axitinib (*n* = 25)	Everolimus + Lenvatinib (*n* = 19)	Bevacizumab (*n* = 17)	Ipilimumab + Nivolumab (*n* = 16)	Axitinib + Pembrolizumab (*n* = 13)	Pazopanib (*n* = 12)
Real-world progression-free survival							
Median (95% CI)	7.0 (5.1, 8.7)	3.6 (2.8, 5.3)	6.7 (3.6, 9.6)	6.5 (1.7, 14.6)	4.9 (2.3, 7.8)	8.1 (2.8, 11.2)	6.9 (3.6, 18.6)
12-month PFS probability, % (95% CI)	31.5% (22.0, 41.4)	16.9% (4.6, 35.7)	16.8% (4.2, 36.8)	29.4% (10.7, 51.1)	25.0% (7.8, 47.2)	17.3% (2.8, 42.4)	45.5% (16.7, 70.7)
Multivariable analysis, Adjusted HR	Reference	1.79 (1.02–3.15)	1.19 (0.66–2.16)	1.14 (0.67–1.95)	1.18 (0.71–1.96)	1.69 (0.97–2.92)	0.98 (0.54–1.77)
Overall survival							
Median (95% CI)	19.5 (16.4, 26.9)	15.7 (5.2, 19.1)	17.8 (3.9, 35.8)	30.3 (11.3, NR)	10.0 (2.6, 19.7)	13.5 (6.9, NR)	20.7 (8.9, NR)
12-month OS probability, % (95% CI)	73.4% (62.7, 81.5)	56.4% (33.8, 74.0)	55.9% (30.8, 75.0)	70.6% (43.1, 86.6)	46.4% (20.4, 69.0)	50.8% (21.4, 74.2)	81.8% (44.7, 95.1)
Multivariable analysis, Adjusted HR	Reference	1.44 (0.75–2.76)	1.08 (0.37–3.15)	0.62 (0.27–1.42)	1.63 (0.85–3.14)	1.77 (0.83–3.76)	1.12 (0.55–2.27)

Abbreviations: CI—confidence interval; KM—Kaplan–Meier; LOT—line of therapy; NA—not applicable; NR—not reached; OS—overall survival; and rwPFS—progression-free survival.

## Data Availability

Per IRB exempt determination/waiver of authorization and consent, all patient-level data are de-identified in accordance with HIPAA Section 164.514 (b) and (c). The data that support the findings of this study are available from Ontada, a McKesson Corporation business, but restrictions apply to the availability of these data due to the risk of reidentification. We are unable to share these data outside the organization.

## Supplementary Materials

The following supporting information can be downloaded at https://www.mdpi.com/article/10.3390/cancers17091434/s1, Table S1: Association of individual index treatments with clinical outcomes: A results from Cox proportional hazards regression model.
