# Supplementary material for: Real-World Treatment Patterns and Clinical Outcomes Among Patients with Metastatic Renal Cell Carcinoma Post-Immune-Oncology and Vascular Endothelial Growth Factor Receptor Targeted Therapies
Source: Cancers (Basel). 2025 Apr 25;17(9):1434. doi: 10.3390/cancers17091434 (PMC12071004; doi:10.3390/cancers17091434)
Supplement: Supplementary file 1 [file cancers-17-01434-s001.zip › cancers-3492434-supplementary.pdf]

Supplementary Table

Table S1. Association of individual index treatments with clinical outcomes: A results from Cox proportional hazards regression model

Variable	PFS Adjusted HR (95% CI)	P value	OS Adjusted HR (95% CI)	P value
**Individual treatments**				
Cabozantinib (reference)	1 [Reference]		1 [Reference]	
Axitinib	1.79 (1.02-3.15)	0.0420	1.44 (0.75-2.76)	0.2699
Axitinib + Pembrolizumab	1.19 (0.66-2.16)	0.5580	1.08 (0.37-3.15)	0.8906
Bevacizumab	1.14 (0.67-1.95)	0.6380	0.62 (0.27-1.42)	0.2596
Everolimus + Lenvatinib	1.18 (0.71-1.96)	0.5180	1.63 (0.85-3.14)	0.1410
Ipilimumab + Nivolumab	1.69 (0.97-2.92)	0.0624	1.77 (0.83-3.76)	0.1385
Pazopanib	0.98 (0.54-1.77)	0.9337	1.12 (0.55-2.27)	0.7586
Others	1.65 (1.10-2.49)	0.0163	1.01 (0.65-1.59)	0.9553
**Line of therapy**				
LOT2	1 [Reference]		1 [Reference]	
LOT3	1.25 (0.75-2.08)	0.3881	1.32 (0.75-2.33)	0.3364
LOT4+	1.36 (0.74-2.51)	0.3232	1.36 (0.65-2.83)	0.4193
**Age at index**	1.00 (0.99-1.02)	0.4939	1.01 (0.99-1.03)	0.2807
**Gender**				
Male	1 [Reference]		1 [Reference]	
Female	0.65 (0.46-0.91)	0.0136	0.85 (0.57-1.26)	0.4132
**Race**				
White	1 [Reference]		1 [Reference]	
Black	0.62 (0.32-1.20)	0.154	0.95 (0.42-2.17)	0.9048
Other	1.10 (0.75-1.60)	0.6299	1.14 (0.70-1.85)	0.5951
**ECOG**				
0-1	1 [Reference]		1 [Reference]	
2+	1.47 (0.91-2.38)	0.1205	2.27 (1.20-4.28)	0.0114
Not documented	1.20 (0.85-1.69)	0.3104	1.85 (1.24-2.77)	0.0028
**Histology**				
Clear cell	1 [Reference]		1 [Reference]	
Non-clear cell	0.97 (0.61-1.55)	0.9046	1.19 (0.64-2.21)	0.5788
Not documented	1.56 (0.83-2.92)	0.1647	3.03 (1.59-5.79)	0.0008
**IMDC**				
Favorable/Intermediate	1 [Reference]		1 [Reference]	
Intermediate/Poor	1.12 (0.80-1.56)	0.5026	1.21 (0.78-1.87)	0.3967
Not documented	1.13 (0.58-2.20)	0.7104	1.73 (0.98-3.05)	0.0569

Abbreviations: 1L - first-line; BMI - body mass index; CI - confidence interval; ECOG - eastern cooperative oncology group; HR - hazard ratio; IMDC - international metastatic renal cell carcinoma database consortium; IO - immuno-oncology; IQR - interquartile range; mRCC - metastatic renal cell carcinoma; OS - overall survival; PFS - progression-free survival; SD - standard deviation; TKI - tyrosine kinase inhibitors
